# Characteristics of gated treatment using an optical surface imaging and gating system on an Elekta linac

**DOI:** 10.1186/s13014-015-0376-x

**Published:** 2015-03-19

**Authors:** Philipp Freislederer, Michael Reiner, Winfried Hoischen, Anton Quanz, Christian Heinz, Franziska Walter, Claus Belka, Matthias Soehn

**Affiliations:** Department of Radiation Oncology, LMU University Hospital, D-81377 Munich, Germany

**Keywords:** Respiratory gating, Catalyst, Latency, Dosimetry

## Abstract

**Background:**

Knowing the technical characteristics of gated radiotherapy equipment is crucial for ensuring precise and accurate treatment when using techniques such as Deep-Inspiration Breath-Hold and gating under free breathing. With one of the first installations of the novel surface imaging system Catalyst™ (C-RAD AB, Sweden) in connection with an Elekta Synergy linear accelerator (Elekta AB, Sweden) via the Elekta Response Interface, characteristics like dose delivery accuracy and time delay were investigated prior to clinical implementation of gated treatments in our institution.

**Methods:**

In this study a moving phantom was used to simulate respiratory motion which was registered by the Catalyst™ system. The gating level was set manually. Within this gating window a trigger signal is automatically sent to the linac initiating treatment delivery. Dose measurements of gated linac treatment beams with different gating levels were recorded with a static 2D-Diode Array (MapCheck2, Sun Nuclear Co., USA) and compared to ungated reference measurements for different field sizes. In addition, the time delay of gated treatment beams was measured using radiographic film.

**Results:**

The difference in dose delivery between gated and ungated treatment decreases with the size of the chosen gating level. For clinically relevant gating levels of about 30%, the differences in dose delivery accuracy remain below 1%. In comparison with other system configurations in literature, the beam-on time delay shows a large deviation of 851 ms ± 100 ms.

**Conclusions:**

When performing gated treatment, especially for free-breathing gating, factors as time delay and dose delivery have to be evaluated regularly in terms of a quality assurance process. Once these parameters are known they can be accounted and compensated for, e.g. by adjusting the pre-selected gating level or the internal target volume margins and by using prediction algorithms for breathing curves. The usage of prediction algorithms becomes inevitable with the high beam-on time delay which is reported here.

## Background

Respiratory motion is still one of the major sources for uncertainties in thoracic and abdominal treatment sites in radiation therapy. The accuracy of dose delivery can be increased by respiratory-adapted gating or breathing control [1]. Accounting for intrafraction motion solely by increasing the treatment margins will increase the volume of normal tissue being irradiated with high doses [2]. Therefore techniques to minimize treatment margins are highly desirable. With the introduction of gated treatments, in which the beam is only activated during specific motion phases (the so-called gating window), [3,4] the increased organ-at-risk (OAR) dose can potentially be reduced to a minimum. Gated treatment has the potential to reduce lung dose for the radiotherapy of thoracic esophageal carcinoma using Deep-Inspiration Breath-Hold (DIBH) techniques [5] and it is considered reliable and effective for patients with high tumor movements in stereotactic body radiotherapy (SBRT) [6]. In order to ensure precise and accurate treatment several gating characteristics, including dose delivery accuracy, overall latency, and temporal accuracy of the applied system have to be known in advance [7]. Gating is initiated with different respiratory monitoring systems at the moment and various studies have been performed in order to assess the differences between them [8-11]. Several others additionally investigated the question whether or not a linac is able to being gated, [3] or the linac’s performance under gated treatment [12-14].

So far the novel surface imaging system Catalyst™ (C-RAD AB, Uppsala, Sweden) in connection with an Elekta Synergy linear accelerator (linac) with Agility Head (Elekta AB, Stockholm, Sweden) and connectivity between those two systems via the Response™ gating interface (Elekta AB, Stockholm, Sweden) has only been examined by calculating theoretical time delays for this system configuration [15]. Dose delivery and time delay have already been measured for other vendors. However, the characteristics of this specific system setup are not known yet. Technically, the gated delivery is being performed by the Response™ interface, which interrupts the RF source during the beam-off period [16]. Our main focus in this study is therefore not only the time delay of the optical surface scanner for the treatment initiation, but the overall system latency, which includes all parts that could possibly delay beam initiation.

## Methods

**Figure 1:**
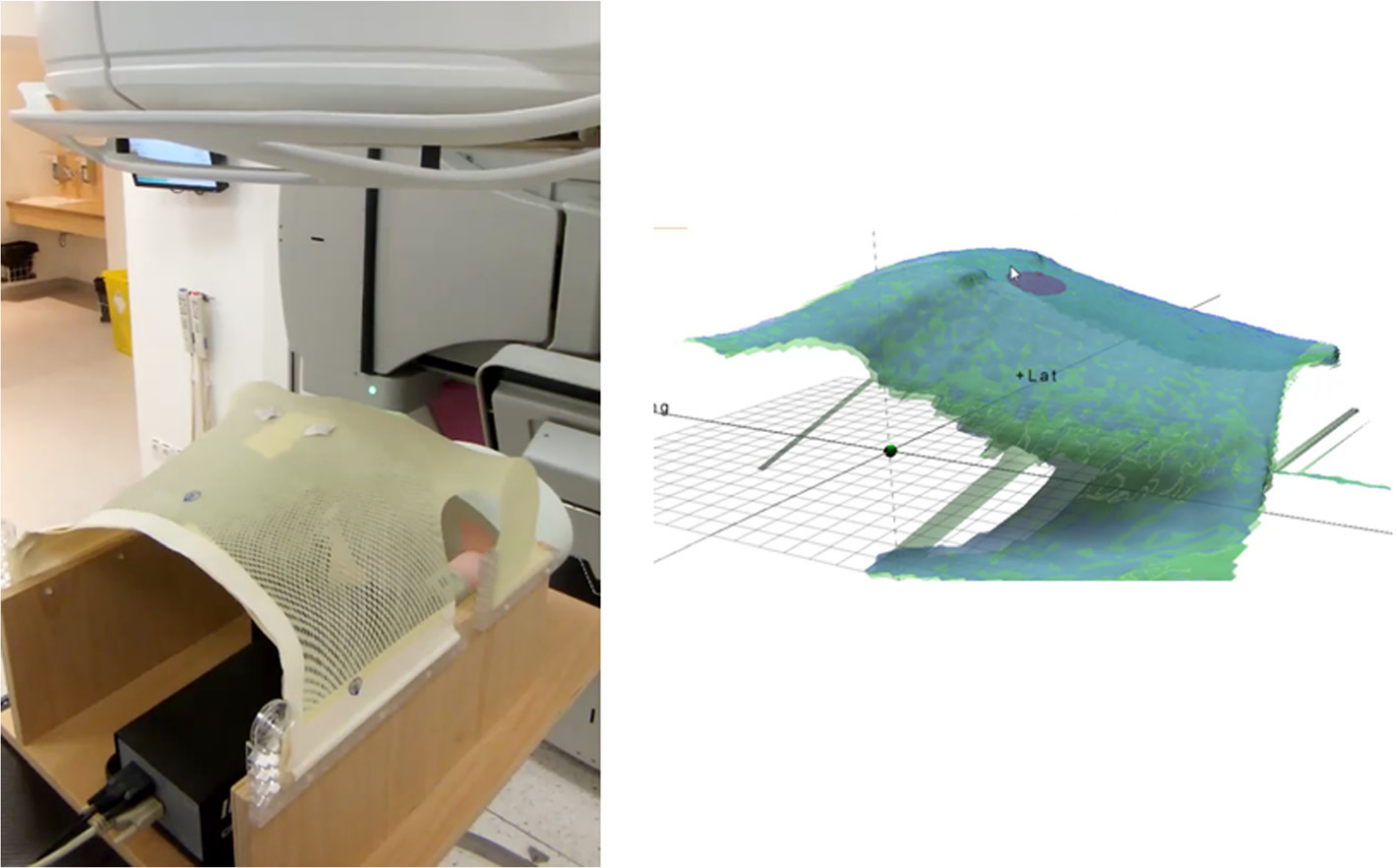
**Left: Image of the build-up for the dynamic moving phantom.** **The surface is reconstructed using mask material. Right: The phantom as it is perceived by the Catalyst™ software.**

In this study, the gating signal used for the linac is delivered by a surface imaging system. A Dynamic Thorax Phantom (CIRS Inc., Norfolk, VA, USA) was used to simulate a breathing waveform, which is then measured by the Catalyst™ system. One of the first steps of integrating a system based on optical surface measurements was to extend our movement phantom (Figure 1, left). There was a need to construct a surface which can be detected by the surface scanner and reproduces breathing patterns imitating natural physiology. Thermoplastic mask material was mounted to the vertical motion platform of the phantom to mimic a human thorax and to create a surface visible for the scanner.

After defining a measurement point on the phantom surface, the vertical movement is recorded by the commercially available Catalyst™ software. The gating level, which in contrast to other commercially available monitoring systems is a spatial gating window in a specific millimeter range, is set manually. Whenever the point of measurement is detected within the gating level a trigger signal is sent automatically to the linac, initiating treatment delivery.

### Dose measurements

In order to measure possible dose differences induced by gated treatment a 2D-Diode Array (MapCheck2, Sun Nuclear Co., Melbourne, FL, USA) was set up stationary. Ungated dose delivery was defined as reference and the absolute dose of the delivery using several gating levels was compared against this reference dose. The array was placed isocentric in SSD = 100 cm and gating levels were chosen as 50%, 40%, 30%, 20%, and 10% of a modulated sine wave simulated by the moving phantom and a total of 300 monitor units (MU) per measurement were applied. Measurements were repeated three times for each gating level for two different field sizes (10 × 10 cm^2^ and 20 × 20 cm^2^).

### Time delay measurements

**Figure 2:**
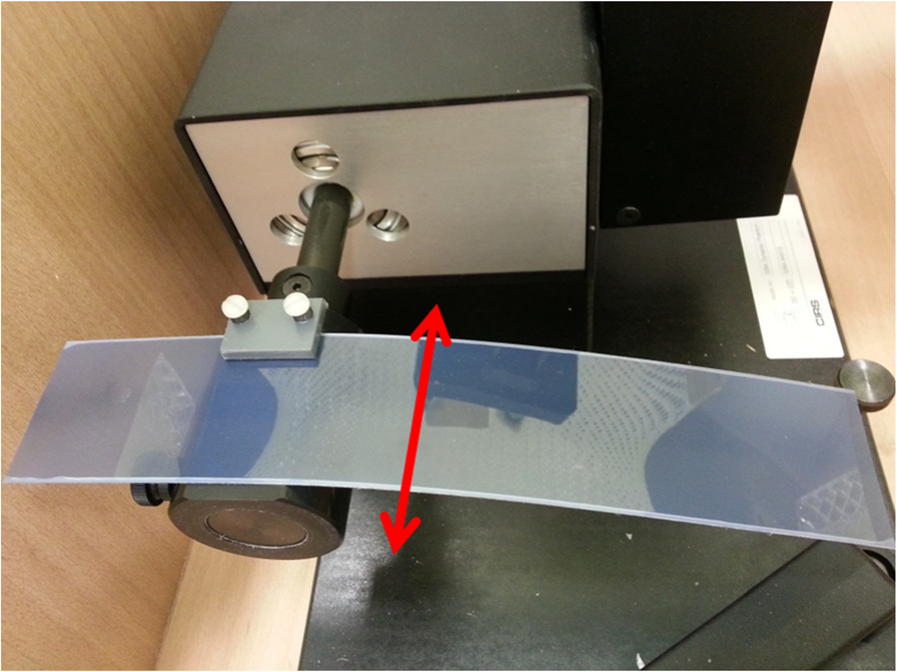
**The custom-built extension for the film measurements.** Here the extension is placed on the moving rod of the phantom and is moved only in the horizontal direction (red arrow).

**Figure 3:**
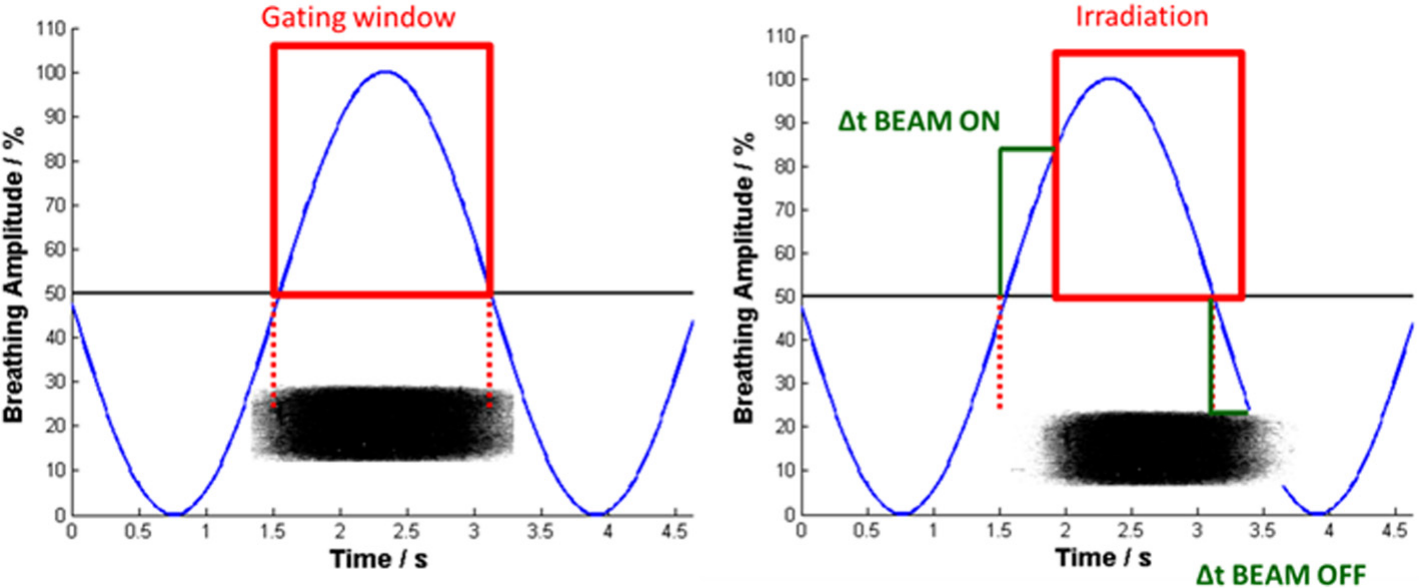
**Left: Ideal case with the absence of time delay.** The blackening of the film on the bottom has the exact length of the desired irradiation. **Right: Real case with system latency**: There is a difference in exposure length between the BEAM-ON signal and the actual exposure of the film (*ΔL*_*BEAM-ON*_) and a difference between the termination signal of the linac and the actual termination of the beam (*ΔL*_*BEAM-OFF*_). Both of these can be measured and with a given velocity, the time delay of the system (*Δt*_*BEAM-ON*_ and *Δt*_*BEAM-OFF*_) can therefore be calculated. The schematic blackening of the film on the bottom of each subfigure is a transposition of the film onto the time axis, as *ΔL* behaves linear proportional to the time delay *Δt* for given velocities.

An extension to the existing phantom for the application of radiographic films (Gafchromic® EBT, International Specialty Products, Wayne, NJ, USA) was built in order to move the film horizontally through the gated beam (Figure 2). As the film moves through a rectangular field with 2 cm × 2 cm field size with 6 cm peak-to-peak amplitude (sinusoidal motion trajectory), blackening of the film due to irradiation is expected in a 2 cm by 5 cm rectangle (2 cm in width and height due to the field size and an additional 3 cm in height due to the gating window of 50% of the film movement) in an ideal case of no time delay in the overall system. The gating level was set to 50% (see Figure 3) for sinusoidal film movements, as at this level the film is moving with minimal acceleration and constant velocity. With a blackening of the film exceeding the ideal (“no time delay”) blackening with a certain length (*ΔL*) for beam off time delay (*Δt*_*BEAM-OFF*_) and vice versa for beam on time delay (*Δt*_*BEAM-ON*_), these two measures can by calculated for the known velocity (*v*) of the film at this level by *Δt = ΔL / v*. Figure 3 shows the schematic measurement principle. For a more detailed description of principle of the methods see Smith & Becker [8]. The overall time delay is calculated as a mean value from in total 6 measurements for each beam-on and beam-off time delay. The blackening of the film and consequently the length *L* was chosen as the part where it has reached its maximum intensity, which is equivalent to the linac reaching its maximum dose rate.

## Results

### Dose measurements

Measured differences in dose delivery between gated and ungated treatment averaged over the whole field size can be seen in Table 1. The total delivered dose decreases with the size of the gating window. With a gating window of only 10% of the breathing cycle in a 10 × 10 cm^2^ field, a dose reduction of 2.15 ± 0.05% can be observed. In addition, the treatment time for such a gating window as narrow as 10% would be increased by a factor 10 in comparison with ungated treatment. For a larger field size of 20 × 20 cm^2^ a dose reduction of 1.62 ± 0.05% is measured in contrast to ungated treatment. The absolute number of start-up processes of the linac are naturally dramatically increased when reducing the gating window while maintaining the same amount of MUs. The relationship between dose uncertainties and the number of start-up processes (in our case about 70–90 using a 10% gating window and about 5–7 with a 50% gating window) becomes obvious. For larger (and therefore temporally longer) gating windows which are window sizes with more practical and clinical relevance, the differences in dose delivery accuracy decrease below 1%.

**Table 1:** **Results of the dose measurements for two different field sizes: for both field sizes, there is a decrease in dose relative to ungated treatment when reducing the gating level and therefore increasing the number of start-up processes and delivery time**

**Gating level**	**Relative dose (10 x 10 cm** ^**2**^ **field)**	**Relative dose (20 x 20 cm** ^**2**^ **field)**
50%	99.41 ± 0.07%	99.59 ± 0.05%
40%	99.43 ± 0.04%	99.53 ± 0.04%
30%	99.45 ± 0.52%	99.46 ± 0.04%
20%	98.81 ± 0.05%	99.09 ± 0.05%
10%	97.85 ± 0.04%	98.28 ± 0.05%

### Time delay measurements

We found a value of *Δt*_*BEAM-OFF*_ = 215 ± 69 ms for the system latency for beam off. For the latency of the beam-on time, however, a value of *Δt*_*BEAM-ON*_ = 851 ± 100 ms has been measured, which is in contrast to current literature, in which delays smaller than 300 ms have been reported [16]. With the current setup for film measurements, the relatively high standard deviation of 100 ms is explainable through measurement uncertainties due to the resolution of the film, the scanning procedure, and the determination of the starting point of *ΔL*. It is crucial to define the timepoint (or point on the film respectively), that allows for a clear blackening of the film which occurs when the linac has reached its maximum dose rate. The dose rate varies uncontrollably during each start-up procedure of the linac due to its transient response, which can be seen as a reason for the high time delay.

## Discussion

The dose delivery accuracy is comparable to current literature. Evans et al. reported dosimetric differences below 1% for beam-on times higher than 0.5 s with a comparable linac from the same manufacturer [16]. In our case, this beam-on-period would be comparable to a gating window of about 30% where the difference in dose has also been measured below 1%. Even when regarding different linac vendors, dosimetric differences stay constant at around the same level, although only larger gating windows have been evaluated [3]. A direct comparison of respiratory monitoring systems is not possible, since most of these have been evaluated with Varian linacs, [9,11] or for proton treatment sites [4]. However the data found in literature is not comparable to the findings in this study as Varian uses a different gating approach. For example, the BrainLAB ExacTrac gating system (BrainLAB, Feldkirchen, Germany) in combination with the Varian real-time position management (RPM) gating system (Varian Medical Systems, Palo Alto, CA, USA) were found to have a tracking time delay for the monitoring system only of 200 ± 30 ms and 90 ± 10 ms time delay for beam-on and beam-off times respectively, but there is no mention about the start-up process of the linac afterwards and the subsequent dose delivery to the patient [10].

The method for measuring the system latency has been adapted from Smith & Becker [8]. This particular method proves to be efficient and accurate enough in order to measure the overall time delay. Another method has recently been proposed by Cui et al., [15] which, however, does not measure the time delay for each start-up process, does not incorporate the beam-off delay, and the variable dose rate cannot be distinguished in their measurements. In our measurement setup, the time when dose rate variations occur during each start-up process are not considered as beam-on. However the investigators propose options for the optimization of this issue by changing certain parameters of the linac, such as the gun hold-on time (GHT), which is by default at a level of 1.38 s. Increasing this parameter causes the electron gun to stay in an active state rather than switching to standby mode. Once the electron gun reaches this standby mode, it consequently takes longer to be in a stabilized active mode again [15]. This is an interesting point which is to be determined in the future at our site, as also further dosimetric measurements have to be performed. However, increasing the GHT could result in a lower life time of the electron gun and is a setup which has to be measured in a different way. Up to this point only DIBH techniques are implemented at our site, hence the lower GHT is to be considered as the regular system setup.

Besides others, two main effects contribute to the overall system latency, composed by the time delay of the surrogate system (in our case the optical surface scanner) and the time delay of the linac itself. The rather high beam-on time delay of about 850 ms reported here is expected to have its source primarily in the time delay of the linac. According to Lund University [priv. comm.], the mean time delay of the surrogate alone has been measured with about 162 ms for beam-on time and 262 ms for beam-off time using a pneumatic piston in order to generate the breathing pattern. They have measured the time between the output of a trigger signal and the change in piston position digitally, which could not be performed in our site, but would be a valid way of determining the latency of the surrogate system alone. The beam-off delay of 262 ms for the surrogate system alone is higher than the beam-off delay for the whole treatment chain measured here as we were able to perform our measurements with a newer software version of the surrogate system.

The difference in dose and the time delay for beam-on are due to the fact, that every start-up process of the linac is accompanied by a particular uncertainty because of the linac’s transient response. The choice of the duty cycle is crucial: With a decrease in dose of about 2% at the 10% duty cycle and an increased treatment time, awareness is needed when deciding on how far margins should be reduced through gated treatment: There will always be a trade-off between choosing a smaller gating window to reduce margins as far as possible, which reduces residual geometric errors against having this smaller gating window enhance possible dosimetric errors.

Predictive algorithms for respiratory motion implemented in the software of the optical surface scanner could potentially compensate for errors caused by time delays. Up to this point, such algorithms can predict respiratory motion up to 1000 ms [17]. Of course, the quality of these prediction methods is still limited up to a certain extent and larger time delays, as they have been measured here, will also be harder to compensate for, even with a prediction of respiratory motion. A detailed overview of different prediction models and approaches can be found in [18]. As interfractionally both the tumor position and the gating window can change throughout the course of the entire treatment, [19] a periodical update for these will also be required.

Once parameters like time delay and dose distribution are known, they have to be accounted for and compensated by for example adjusting the pre-selected gating level or the internal target volume (or also the clinical target volume, CTV) margins.

## Conclusions

When performing gated treatments, especially free-breathing gating, it is crucial that factors such as time delay and dose delivery accuracy have to be determined in advance. In addition regularly QA-measurements as proposed by the AAPM Task Group 76 [2] need to be performed in order to assure stability over time. Our data also indicates the need for the usage of predictive algorithms describing the breathing curves whenever the curves are finally used for gated treatments.

The examined system setup can and is being used for techniques such as DIBH, where a high time delay of about 850 ms is automatically compensated up to a certain extend due to longer gating cycles with less start-up processes of the linac. When it comes to free-breathing gating, certainly some parameter changes (such as the GHT) in the linac and the proposed prediction methods are essential.
